# Soft Epidermal Paperfluidics for Sweat Analysis by Ratiometric Raman Spectroscopy

**DOI:** 10.3390/bios14010012

**Published:** 2023-12-25

**Authors:** Ata Golparvar, Lucie Thenot, Assim Boukhayma, Sandro Carrara

**Affiliations:** 1 Bio/CMOS Interfaces (BCI) Laboratory, École Polytechnique Fédérale de Lausanne (EPFL), 2000 Neuchatel, Switzerland; 2 Senbiosys SA, 2000 Neuchatel, Switzerland

**Keywords:** cellulose-based sensors, eco-friendly, non-invasive, optical biosensors, skin-interfaced biosensors, sustainable development, sweat analysis, sweat loss, wearable

## Abstract

The expanding interest in digital biomarker analysis focused on non-invasive human bodily fluids, such as sweat, highlights the pressing need for easily manufactured and highly efficient soft lab-on-skin solutions. Here, we report, for the first time, the integration of microfluidic paper-based devices (μPAD) and non-enhanced Raman-scattering-enabled optical biochemical sensing (Raman biosensing). Their integration merges the enormous benefits of μPAD, with high potential for commercialization and use in resource-limited settings, with biorecognition-element-free (but highly selective) optical Raman biosensing. The introduced thin (0.36 mm), ultra-lightweight (0.19 g), and compact footprint (3 cm^2^) opto-paperfluidic sweat patch is flexible, stretchable, and conforms, irritation-free, to hairless or minimally haired body regions to enable swift sweat collection. As a great advantage, this new bio-chemical sensory system excels through its absence of onboard biorecognition elements (bioreceptor-free) and omission of plasmonic nanomaterials. The proposed easy fabrication process is adaptable to mass production by following a fully sustainable and cost-effective process utilizing only basic tools by avoiding typically employed printing or laser patterning. Furthermore, efficient collection and transportation of precise sweat volumes, driven exclusively by the wicking properties of porous materials, shows high efficiency in liquid transportation and reduces biosensing latency by a factor of 5 compared to state-of-the-art epidermal microfluidics. The proposed unit enables electronic chip-free and imaging-less visual sweat loss quantification as well as optical biochemical analysis when coupled with Raman spectroscopy. We investigated the multimodal quantification of sweat urea and lactate levels ex vivo (with syntactic sweat including +30 sweat analytes on porcine skin) and achieved a linear dynamic range from 0 to 100 mmol/L during fully dynamic continuous flow characterization.

## 1. Introduction

Digital health has witnessed significant growth over the past few years, owing to the introduction of wearable electronic devices capable of sensing a diverse array of biosignals, including those related to cardiac, neural, ocular, and musculoskeletal functions [1,2,3]. These wearables, when coupled with established sensing modalities for measuring parameters such as heart rate, blood oxygenation, and body temperature, as well as signals indicative of motion, strain, stiffness, and electrical impedance, have facilitated the development of highly advanced remote healthcare systems [4,5]. As a significant improvement, nowadays, these systems are becoming increasingly compact and socially pervasive [6]. Nonetheless, these almost matured systems primarily capture macroscopic manifestations of physiological processes, offering limited insight into the underlying etiology of health issues [7]. 

Consequently, accurate and personalized diagnosis, prediction, and management of various diseases remain challenging in point-of-care testing (POCT) settings. To address this limitation, the next generation of wearable devices is poised to undertake molecular analysis from dermal biofluids such as sweat and interstitial fluid [8,9]. 

Eccrine sweat, due to its easy and non-invasive accessibility through numerous sweat glands, is of particular interest for assessing the biochemistry of the human body. Sweat contains exogenous non-metabolic agents and metabolites, proteins, electrolytes, and hormones, often with high clinical value, and is a potential probe to quantify biological age [10]. On the other hand, sweat analysis has extensive applications, from drug monitoring and therapeutic dosing [11], to quantifying essential nutrients, vitamins, and minerals like vitamin C, calcium, zinc, and iron [12]. For instance, sweat chloride monitoring is well-established for diagnosing cystic fibrosis [13]. Furthermore, sweat glucose may be used for diabetes management [14], and sweat uric acid for gout monitoring [15]. Mostly, the aforementioned sweat analysis applications rely on smart patches that are soft, ultrathin, and ultra-lightweight. Typically, these patches are meticulously engineered to establish seamless skin interaction and are enclosed in materials that mimic the epidermal layer, facilitating the sensing process while ensuring a conformal and secure connection with the skin surface. At their core, they consist of two primary components: a sweat collection and transportation section, “micro/paper fluidic”, and a bio-chemical analysis compartment, “bio/nano interface”.

However, these soft “micro/paper fluidic” platforms require complex fluidic design and additional surface engineering to facilitate passive sweat-gland-driven pumping. In addition, fabricating those also usually relies on extensive processes. On the other hand, those “bio/nano interfaces” primarily employ colorimetry, electrochemical, or field-effect transistor-based biosensing modalities, typically functionalized or immobilized with various biorecognition or bioreceptor elements on their sensing spot to enable the selective detection of specific sweat biomarkers [16]. However, typically acquired enzyme-, ionophore-, and antibody-based (immunosensors) recognition elements are susceptible to degradation over time and tend to lose their functionality when exposed to harsh environmental conditions or contamination [17,18]. In addition, the production of enzymes and antibodies for biosensors is resource-intensive and involves ethically challenging practices, especially in the case of antibody production, which often necessitates the use of animals [19]. Moreover, their limited shelf life and energy-intensive procedures required to maintain optimal temperature and pH conditions introduce logistical challenges regarding transportation and storage. Furthermore, the issues related to cross-reactivity with similar molecules further complicate the achievement of high specificity, mainly when dealing with complex sample matrices [20]. Again, despite numerous advancements, biosensing applications employing bioreagent elements stay single-use consumables like disposable sensor strips, leading to waste generation concerns and contributing to the overall cost of biosensing applications. 

One promising avenue for addressing the challenges outlined above concerning microfluidic platforms is the utilization of cellulose-based substrates (i.e., “lab-on-paper”), which presents several significant advantages [21]. Adopting paper as a substitute for conventional microfluidic systems has attracted considerable attention [22]. Firstly, porous materials have remarkable wettability (i.e., liquid absorption) and transport capabilities (i.e., wicking), resulting in instantaneous sweat uptake upon secretion and immediate transportation to the biosensing spot before sweat evaporation begins. Indeed, upon liquid contact with the porous surfaces, it tends to wet that surface, allowing for the subsequent kickstarting of highly efficient spontaneous wicking due to negative capillary pressure occurs in porous media, which is an assemblage of micro/nano-sized capillary tubes [23]. This attribute dramatically reduces system response time, facilitating swift sweat component analysis without the hindrance of evaporation, owing to the rapidity of the transport process and the high accuracy of sweat biosensing due to avoidance of sweat evaporation. 

Indeed, open-surface paperfluidics will still give accurate biosensing results due to the slower evaporation rate of sweat compared to the swiftness of sweat transportation to the biosensing spot. However, open-surface paperfluidics will have erroneous sweat rate analysis based on the wicking rate due to the non-neglectable evaporation effect. However, sweat evaporation from the channel can be minimized to make its effect neglectable by including an encapsulator in the paperfluidics and creating a closed-channel paperfluidics. Secondly, fully sustainable and cost-effective fabrication processes are possible for developing paperfluidic platforms. Furthermore, these soft microfluidic devices are readily biodegradable with existing recycling processes without requiring novel recycling cycle investigations. 

On the other hand, concerning the challenges of using biorecognition elements in the “bio/nano interfaces”, recent strides in microtechnology have enabled the downsizing of conventional laboratory-grade label-free biosensing instruments to be suitable for point-of-care diagnostics [24]. Notable among these advancements is the emergence of optical Raman-scattering-based biochemical sensing (Raman biosensing) as a rapidly evolving approach in remote healthcare, offering a compelling alternative to biorecognition-based biosensing methodologies [25,26]. Raman biosensing capitalizes on its inherent selectivity by exploiting the distinct chemical signatures of biomolecules to generate unparalleled characteristic fingerprint spectra [27,28]. Compared to traditional biosensing techniques reliant on biorecognition elements, in our earlier study, we have shown that Raman biosensing delivers superior performance marked by label-free monitoring capabilities, stable recording, high accuracy, and remarkable long-term operation capability in 90-day long experimentation in multiplexed biomarker detection without requiring temperature or pH calibration [7]. Furthermore, the ongoing pursuit of further miniaturization of portable/handheld Raman systems and their translation to wearable or on-chip systems is now a prevailing focus [29]. This quest encompasses the development of on-chip spectrometers employing innovative planner optics devices [30], bolstered by novel instrumentation methodologies such as “single-band Raman analysis” and “spectroscopy-less Raman biosensing” techniques [29,31]. 

On the other hand, although powerful, spontaneous Raman spectroscopy has been labeled as “not sensitive enough” for quantitative analysis in the past. Consequently, researchers have proposed Raman enhancement techniques, such as surface-enhancement or coherent Raman enhancement methods (e.g., SRS) [32,33]. Indeed, a few studies have already reported the integration of paper-based microfluidic and surface-enhanced Raman spectroscopy (SERS) for not only the detection of micromolar range (or lower) sweat analytes such as uric acid or various drugs but also to monitor micromolar range concentrated sweat analytes such as urea and lactate [17,34,35,36]. Nevertheless, substantial hurdles persist in translating these technologies into practical applications. Coherent Raman techniques face constraints related to their reliance on femtosecond laser technology, which poses challenges for miniaturization within the current technological landscape [32]. On the other hand, developing plasmonic surfaces for SERS is hindered by low reproducibility due to high optical losses because of absorption in the metal, which severely limits its real-world applications [37]. In addition, current SERS methods come with almost all the limitations encountered in biorecognition-element-based biosensing, such as limited reusability and restricted shelf-life, which challenges favoring SERS over well-established electrochemical biosensors. 

Alternatively, this study presents a novel approach, demonstrating that spontaneous Raman scattering, without enhancing techniques, can quantitatively measure at least two critical sweat biomarkers—sweat urea and lactate, directly in situ after being wicked by a paperfluidic platform (see Supplementary Materials Note S1: Significance of Sweat Urea and Lactate Analysis). Consequently, this study represents the first integration of microfluidic paper-based devices (μPAD) and Raman biosensing without encompassing plasmonic nanostructures. 

Indeed, our study demonstrates that the application of the enhancement methodologies in Raman spectrometry for the analysis of sweat urea and lactate is not necessary, and spontaneous Raman spectroscopy exhibits adequate sensitivity for detecting these analytes within their native fluid environment in their pathophysiological concentration ranges of interest. We then elucidate the straightforward development process of cellulose paper-based epidermal soft optofluidics for rapid sweat collection and transportation. Subsequently, we report an effective methodology for quantifying sweat loss solely through visual analysis, free from acquiring electronic components or imaging techniques. Finally, we demonstrate selective sweat urea and lactate biosensing using ratiometric Raman spectroscopy in fully dynamic in-flow ex vivo experimentations with our “Raman–paperfluidic” system.

## 2. Methodology

The obtained materials followed procedures for human sweat collection, in vitro and ex vivo Raman scattering measurements, and used instruments and implemented data analysis methods described in Supplementary Materials Note S2: Materials and Methods.

### Soft Paper-Based Optofluidic Device: Design, FEM Simulation, and Fabrication 

The design of the channel pattern is inspired by prior investigations that have established the high efficacy of curvilinear shapes in mitigating mechanical deformations [38,39]. As a result, a small footprint (15 mm to 20 mm) serpentine-like pattern was conceptualized and subsequently optimized through finite element method (FEM) simulations employing COMSOL multiphysics to enhance the mechanical resilience of the structure during natural tissue elongation. The properties employed for simulating the channel-layer material, constructed from a cellulose-based paper, encompass an elastic modulus (E) of 420 MPa, density (ρ) of 258 kg/m^3^, and a Poisson ratio of 0.35. The authenticity of these parameters has been previously substantiated through empirical analysis [40]. In addition, the skin model incorporates properties as follows: E = 101.20 kPa, ρ = 1020 kg/m^3^, and a Poisson’s ratio of 0.5, which are also experimentally derived previously [41]. The applied strain on the skin was 23% in both directions, which was again experimentally determined to correlate with the average Lagrange strains exhibited in the skin surface and sub-surface layer during natural tissue deformation [42].

Several process flows exist to fabricate soft microfluidic devices, such as lithography, laser patterning, and stencil printing [43,44]. In this study, we follow a low-cost, super simple, and sustainable alternative fabrication process. Indeed, the wearable paper-based epidermal soft optofluidics (i.e., epi-opto-paper fluidics) presented herein only comprises a cellulose-based channel layer sandwiched between an adhesive and an encapsulating layer. A demonstrative video recording illustrates the straightforward development of the device (Supplementary Video SI, min:sec: 00:00 until 04:30). The first layer of the unit consists of a double-sided medical-grade silicone/acrylate thermoplastic elastomer tape (2477P, 3M, USA), serving a dual purpose. Firstly, it ensures a conformal attachment of the paperfluidic unit to the epidermis, ensuring secure and intimate contact without irritation. Secondly, this adhesive layer plays a crucial role in maintaining the structural integrity of all constituent layers within the paperfluidic unit. Indeed, all other layers are affixed to the device via attachment to the first layer. In applications necessitating high laser powers, a laser blocker (T205-1.0—AT205 Black Aluminum Foil Tape, Thorlabs, USA) is included in the fabrication process, which is directly attached to the first layer beneath the Raman laser focus spot. This precautionary measure effectively shields the skin from direct exposure to laser radiation, thus mitigating potential hazardous consequences. The intermediate layer comprises 180 μm thick Whatman grade 1 qualitative filter paper (Sigma-Aldrich, MilliporeSigma, USA). To pattern the unmodified filter paper, a cost-effective cutting machine (USD 250) was utilized (Cameo 3, Silhouette America, USA). 

The fabrication parameters, encompassing cutting blade speed, depth, and applied force, were fine-tuned through iterative experimentation to achieve precise cuts while minimizing paper tearing. The optimized parameters for patterning Whatman grade 1 filter papers are as follows: applied force—7 g force (i.e., 0.0686 N); penetration depth—3 mm; velocity—10 cm/s; number of passes—1. Alternatively, laser patterning instruments could be employed [45]. However, our commitment to minimizing the carbon footprint of the fabrication process and utilizing accessible, budget-friendly equipment prompted our chosen approach. 

To seal the system, the fabrication of the uppermost polydimethylsiloxane (PDMS) layer has been optioned with a mixture of Sylgard 184 silicone elastomer (DOW, USA) and its curing agent was prepared in a 10:1 ratio. This viscous PDMS base-curing agent blend was mixed in a planetary mixer at 2000 rpm for 2 min, followed by a 2 min degassing step at the same speed. Subsequently, the doctor-blade-assisted casting technique was employed to generate a 100 µm thick PDMS sheet at a blade speed of 2 mm/s on a thin polyethylene terephthalate (PET, MELINEX ST506, Tekra, USA) carrier substrate. The PDMS sheet was cured at 80 °C for 1 h for solidification. A thin PET layer was introduced post-curing to encapsulate the PDMS layer, effectively minimizing the potential accumulation of dust particles on the PDMS surface.

## 3. Results 

### 3.1. Wearable Epidermal Paper-Based Optofluidics 

**Figure 1:**
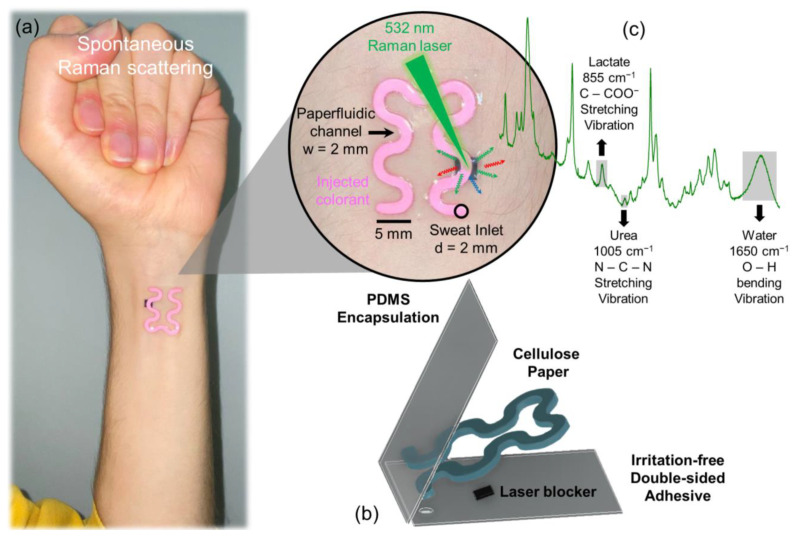
(**a**) The introduced paper-based epidermal soft optofluidic device (referred to as “epi-opto-paper-fluidics”) positioned on the epidermis to facilitate non-invasive and label-free biochemical analysis of sweat components (when coupled with a Raman spectroscopy) and simultaneous visual quantification of sweat loss volume. (**b**) It comprises sequential layers: a PDMS encapsulator layer atop, a precisely patterned cellulose paper-based channel layer at the core, an integrated, flexible laser blocker tape, and an underlying adhesive layer. (**c**) The resulting congested Raman scattering spectra are dominated by cellulose and PDMS-linked Raman shifts. The influence of sweat samples manifested with selective Raman shifts correlated linearly in intensity with concentrations of sweat biomarkers such as lactate and urea. To improve the visualization of the unit, a diluted Rhodamine 6G solution is introduced in the inlet, which is wicked entirely by the paperfluidic changing its color to pink. In reality, the white channel color transitions to grayish upon sweat absorption, like typical wetted paper.

Figure 1a shows the paperfluidic unit as affixed to the skin and its layer-by-layer composition, comprising the PDMS encapsulator, patterned cellulose paper channel, laser blocker tape, and underlying adhesive layer. The induced Raman scattering spectrum highlights selective Raman shifts corresponding to sweat lactate and urea concentrations. The paperfluidic unit, as realized (see Supplementary Figure S1a), can be easily detached from its protective layers (e.g., Supplementary Figure S1b) and attached to the skin (Supplementary Video SI: 4:30 until 5:20). Next, upon gentle application of pressure (Supplementary Figure S1c), the unit conforms to the epidermis (Supplementary Figure S1d) and can effectively endure the inherent stretching of the skin due to its robust mechanical integrity, validated through rigorous skin surface deformation assessments (Supplementary Figure S1e,f). In scenarios necessitating elevated laser power use, strict confinement of laser irradiation to predefined regions housing the laser-blocking layer is required. The potential choices for laser-blocking materials were experientially evaluated (Supplementary Figure S3), and notably, the filter paper itself exhibited remarkable efficacy in blocking 98.5% of incident laser power. As an added precaution, an alternate ~110 μm thick aluminum laser-blocking tap, inherently capable of attenuating 99.77% of laser radiation, was also incorporated to enable near-ideal blockage of laser radiation from reaching the skin. The packaging (Figure 1b), which involves sandwiching the device between a protective layer (positioned beneath the adhesive) and a thin PET sheet (positioned above the PDMS layer), reduces dust accumulation during storage. Therefore, it facilitates mass production of the devices within a single production cycle (100 units in a single day), enabling their utilization throughout this investigation. Notably, we employed units that were stored for a period exceeding six months under standard room conditions, and we observed no changes in their operational efficiency.

**Figure 2:**
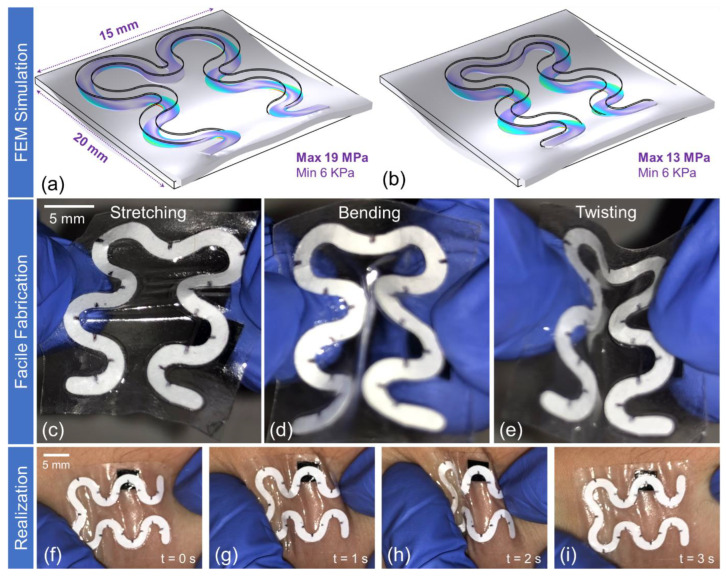
The three phases of developing the introduced soft microfluidic device: design and optimization through mechanical simulations, printing-free facile fabrication using elementary tools, and in situ applications. (**a**) The initial design of the channel layer, (**b**) optimized design directed to enhance mechanical robustness. The evaluation of mechanical deformation is demonstrated through in vitro experiments following the removal of protective packaging: (**c**) involves axial stretching, (**d**) in-plan bending, and (**e**) twisting tests. Subsequently, the mechanical resilience is further investigated post-attachment to a female epidermis over varying temporal frames: (**f**–**i**) visually elucidates the sustained resilience of the device under applied stress. The channel incorporates discrete black markings within its PDMS layer, which serve as markers enabling real-time visualization of fluid front progression. This visualization is harnessed for accurately determining sweat volume intake (sweat loss quantification) to estimate sweat rate.

Figure 2a illustrates the preliminary design of the channel layer, while Figure 2b presents the optimized design through FEM simulations. Compared to its predecessor, the optimized design demonstrated a 30% reduction in stress. This accomplishment is attributed to the strategic pursuit of smoother and more uniform geometries, which enhances the mechanical resilience of the unit against tensile deformation. The mechanical stability of the developed unit was assessed through two experimental scenarios: in vitro and in situ evaluations. In the initial in vitro assessment, the unit underwent deliberate stretching (Figure 2c), bending (Figure 2d), and twisting (Figure 2e) actions. Subsequently, the unit was attached to male and female volunteers, replicating analogous deformations (Figure 2f–i). These comprehensive assessments were captured through video recordings, conclusively demonstrating the mechanical resilience of the unit against the applied forces (Supplementary Video SI, 5:20 until 6:30, and Supplementary Video SII).

The operational mechanism of the developed soft microfluidics is based on the effective sampling of precise volumes of sweat by the wicking propriety of porous materials through capillary forces (Supplementary Materials Note S3: Wicking Rate Simulation and Estimation) without the need for external pumps or pressure [23,46]. Porous hydrophilic materials have interconnected nano/micro-sized interconnected spaces or pores that can absorb and transmit liquids due to capillary forces [47]. In microfluidic devices composed of hydrophobic solid surfaces such as glass, plastics, or other polymers [48], the capillary action is driven by the combination of cohesive forces within the liquid (i.e., molecules attraction) and adhesive forces between the liquid and the material, forming the narrow space that enables the liquid to rise or be drawn against the force of gravity [49]. Indeed, the initial approach involves utilizing inherently porous and hydrophilic materials. However, in contrast, the latter method generates capillary flow within solid surfaces through the meticulous design of microchannel geometries, which naturally yields a slower fluid flow process. In the case of the presented paperfluidic, the circular inlet of the unit (with a diameter of 2 mm corresponding to ~10 sweat glands) establishes a direct interface with sweat pores on the epidermis. This initiates the sweat collection process almost instantaneously, synchronizing with the onset of sweat secretion—a distinctive characteristic of paperfluidics as opposed to conventional microfluidic platforms (Supplementary Video SI, 6:35). Therefore, attributable to this inherent high efficiency of the wicking property of porous materials, sweat collection with paperfluidics attains elevated effectiveness compared to conventionally employed epidermal microfluidics. Indeed, the latter necessitates the orchestrated interplay of much slower capillary forces and relies mainly on the sweat gland pressure to guide sweat samples into the microfluidics channel. Those devices with engineered material surfaces must present higher hydrophilic surfaces and employ complex structures in their design, such as passive valves [50,51]. Therefore, even with much more complex designs, recently reported soft microfluidic units based on PET had documented latency of 1000 s prior to the commencement of biosensing after sweat extraction starts [52]. Similarly, another investigation employing PDMS-based soft microfluidics has reported a latency duration of 300 s [50]. In contrast, the utilization of paper-based soft microfluidic units remarkably averts this delay, achieving the instantaneous transport of perspired fluid to the designated biosensing region occurring in under 1 min (i.e., 5 times faster) after the onset of sweat secretion.

### 3.2. Impact of the Sweat Rate on the Accuracy of Sweat Components Analysis

Understanding sweat secretion rate is fundamental in accurately interpreting sweat composition data [53]. The process of indirectly estimating or directly measuring sweat rate accounts for factors that influence sweat analysis data, such as temperature, humidity, and physical exertion, during calibration procedures. Estimating the sweat rate is complex, entailing intricate laboratory logistics, but it is considered foundational in translating wearable sweat analysis devices to the market [54]. On the other hand, the interrelation between sweat rate and the concentrations of various sweat analytes constitutes an ongoing subject of discussion [54]. This might be attributed to the multifaceted nature of sweat collection, metrological variations, sample handling procedures, and the analytical techniques employed. Additionally, factors like dilution effects, metabolic activity during different sweat secretion processes (e.g., exercise-induced, thermally induced, drug-induced, and stress/anxiety-induced sweat), individual differences, and specific attributes of analytes, including their size and charge, introduce intricacies into the potential connection between sweat rate and analyte concentration. 

The direction and strength of the relationship about whether the sweat rate influences or not the different analyte concentrations still remains an active field of investigation [55,56]. However, prior investigations were always conducted with labeled analytical methods, which makes intra-lab data interpretation difficult. Consequently, we decided to independently study the interplay between sweat rate and two of the sweat components utilizing our label-free optical biosensing method based on Raman spectroscopy. However, it is essential to note that the constrained scope of our current investigation was not intended to definitively address the prevailing uncertainty regarding the interrelation between sweat rate and analyte concentration. Nonetheless, these findings underscore the imperative of comprehending sweat rate dynamics to ensure the precision of sweat component concentration analyses, which is also the first report utilizing Raman spectroscopy in such a context.

**Figure 3:**
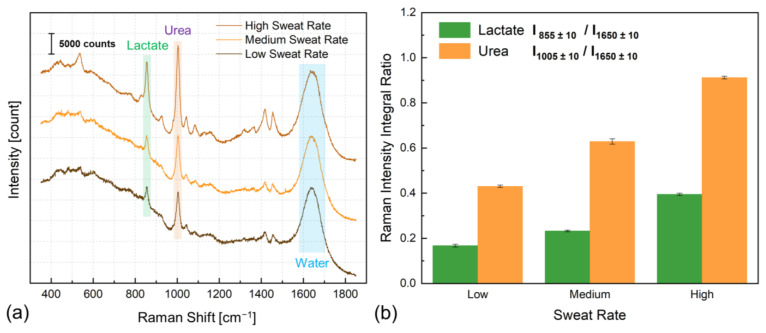
(**a**) Unprocessed Raman spectra (without baseline correction and smoothing) as acquired from human sweat induced by varying exercise intensities, manifesting different sweat rates. Highlighted within these spectra are the shifts corresponding to the characteristic Raman shifts of lactate and urea, positioned at around 855 and 1005 cm^−1^, respectively, and a broad water-related band centered around 1650 cm^−1^. Offsets were added to spectra for visualization. (**b**) The intensity changes in lactate and urea Raman bands, which correspond to the concentration change in sweat collected with diverse sweat rates, reveal the discernible impact of the sweat rate on the analyte concentration.

Figure 3a shows the Raman spectra of sweat induced by exercise at various intensity levels, which resulted in different sweat rate secretion. The Raman spectrum of spontaneous sweat, excluding water, predominantly encompasses lactate and urea [7]. In the most intense exercise-induced sweat spectra depicted in Figure 1a, a minimum of 11 peaks are attributed to lactate (Supplementary Table S1), with the peak at approximately 855 cm^−1^ associated with the C-CO_2_^−^ stretching vibration mode being the most prominent [7]. 

Furthermore, at least one spectrum within this set is assigned to urea, positioned around 1005 cm^−1^, linked to N-C-N stretching vibrations (Supplementary Table S2), and constitutes the most intense urea-linked Raman band. Additionally, the O-H bending mode of water contributes to forming a broad spectral band around 1650 cm^−1^. We observed that the intensity of the water-associated band remains broadly consistent as the sweat rate increases. However, alterations are observed in the intensities of both urea and nearly all lactate-associated peaks. Figure 3b illustrates the intensity ratio of sweat lactate and urea with respect to the Raman shift of water within those sweat samples, which underscores the relevance of sweat rate analysis, particularly in the context of sweat lactate and urea level monitoring.

### 3.3. Chip-Free and Imaging-Less Visual Sweat Rate Estimation

Throughout the years, multiple mechanisms have been described for sweat rate analysis, including both sweat rate “sensing” and sweat rate “estimation” methods [57,58]. For instance, recently, we have shown the possibility of sweat rate sensing in real-time with the flexible printed interdigitated electrode and localized impedance spectroscopy [57]. Similarly, impedimetric sweat rate sensors were incorporated to analyze sweat rate [59]. However, although they may be more accurate, these methods require on-patch electronic circuits and systems. Alternatively, the sweat secretion rate can be estimated by knowing the microfluidics geometry and performing experiments in different conditions to analyze how much liquid in a given duration enters the channel and develop a look-up calibration table [60]. Another more precise method only becomes viable in sedentary sweat collection stimulated by drug delivery (e.g., iontophoresis) or other artificial sweat induction methods where the given amount of drug or electrical current will indicate the sweat rate, and combined with the previous methods, the supposed to be constant sedentary sweat rate can be sensed or estimated [61]. On the other hand, here, we show a chip-free and imaging-less sweat rate estimation method that is based on the wicking property (i.e., liquid update) of porous materials. 

**Figure 4:**
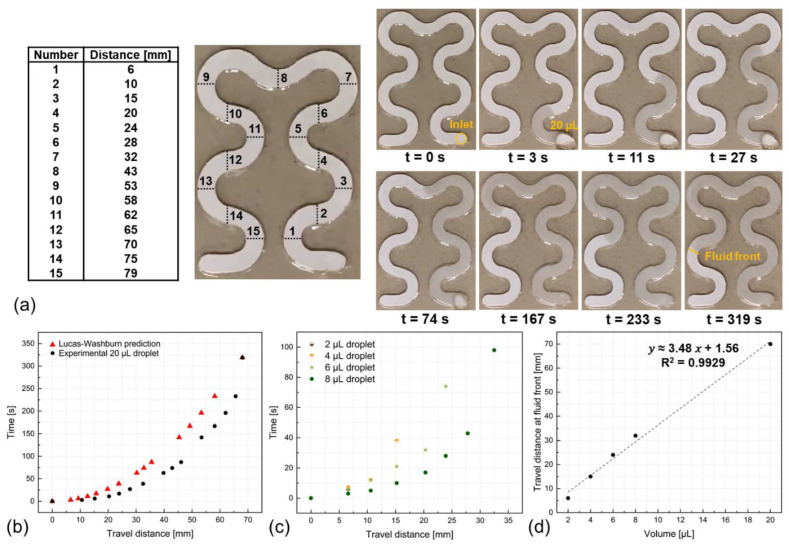
Flow kinetics analysis in the developed paper-based optofluidics. (**a**) Fluid front position over time for 20 μL sweat droplet, which required ~319 s to traverse a distance of ~70 mm. (**b**) The relationship between travel distance and time is examined for a 20 μL droplet within the microfluidic and its theoretical estimation based on Lucas–Washburn equation. (**c**) Travel distance over time for droplets of varying volumes. The investigation spans a range of droplet sizes, effectively highlighting the diverse rates at which these droplets navigate the paperfluidic landscape. (**d**) Highly linear calibration curve for volume uptake using the position of the terminal point of each droplet (fluid front) as shown in (**c**).

Indeed this approch requires lesser calibration requirements compared to previous techniques and is much simpler both in the sense that the physical theory behind it is well known (Lucas–Washburn equation, Supplementary Materials Note S3: Wicking Rate Simulation and Estimation) and its performance is much efficient due to swiftness of liquid uptake by the cellulose paper. To characterize the flow kinetics of the developed paperfluidic to quantify the sweat volume intake and estimate sweat rate, a precise micropipette was employed to place known volumes of artificial sweat droplets onto the channel inlet (Supplementary Video SIII). During calibration, each test involved the use of new microfluidic devices, and five-microliter sample volumes were used to compute the calibration curve, which correlates the travel distance of the fluid within the paperfluidic channel to the volume of the introduced solution, accounting for sweat loss. In this context, sweat loss can be accounted for sweat rate because we can neglect sweat evaporation on the skin since, once secreted, sweat uptake by the paperfluidic inlet is immediate, and the encapsulation layer avoids liquid evaporation during its transportation in the channel. Figure 4a shows the temporal evolution of the travel distance for a 20 μL sweat volume, which reaches its final position in 319 s. The empirically determined wicking rate of this paperfluidic facilitates the determination of sweat loss volume through the observation of fluid front advancement (i.e., travel distance) on the channel, as indicated by discernible alterations in the coloration of the wetted paper. This method permits the visual determination of sweat loss intake without imaging as usually employed [62]. 

This is achieved through the provided mapping table, connecting the position of the fluid front to the effective sweat travel distance and, hence, sweat loss volume (Figure 4a). For instance, for a 20 μL droplet, the fluid front is at about position 13 (Figure 4a). Thus, the sweat travel distance is ~70 mm. Now, if an unknown liquid volume of sweat traverses 70 mm, its volume can be estimated as ~20 μL. Figure 4b shows the temporal behavior of the wicking action of the 20 μL sample in the device. In addition, we have observed a consistent alignment in the behavior between theoretical predictions and experimental findings. This alignment is particularly evident in the behavior of the fluid front (i.e., the actual single data point used in sweat loss quantification), where the last data point shows a perfect congruence between experimental results and theoretical predictions. To investigate the discrepancies further, we explored the impact of a laser blocker, which was found to have a negligible effect. Additionally, introducing a PDMS layer atop the paper marginally accelerated the wicking rate. However, the most significant source of difference was traced back to the assumptions of the governing equation [23]. Unlike the assumption of the Lucas–Washburn equations of continuous flow, our experiments considered the introduction and absorption of limited droplet volume. Furthermore, during the initial few seconds, the wicking speed surpasses the predicted values. This variance can be attributed again to the experimental procedure, where a precise pipette positions the droplet at the inlet. This positioning inevitably exerts a slight force on the droplet, which impacts its initial behavior. 

Similarly, Figure 4c illustrates the travel distances associated with other tested volumes. Indeed, Figure 4d shows the linear dependency of the final data point in Figure 4c (the fluid front position) for each fluid volume with a high R^2^ value of 99.29% and a high sensitivity of 3.48 μL/mm. The experimental scenario with 20 μL volume necessitated 319 s for traversal across 70 mm and culminated in a wicking rate of 3.76 μL/min (standardized to the presented design associated with the porosity of the chosen material and geometry of the designed pattern). As a great advantage, even compared against exceptional sweat rate cases such as the renowned athlete Alberto Salazar, who achieved an extraordinary sweat rate of 3.7 L per hour while training for the 1984 Summer Olympics, the wicking rate of this paperfluidic remains more than this maximum possible human sweat rate. Indeed, for a skin surface area, the maximum possible sweat rate, as mentioned earlier, corresponds to ~370 μL/cm^2^/h, and for a circular area with 2 mm diameter (the inlet of this paperfluidic), this value corresponds to 0.19 μL/min, which is ~19 times lower than the wicking rate of this paperfluidic unit. 

Despite the considered 20 μL volume not constituting the uppermost capacity of the paperfluidic, it is manifest that this rate overwhelmingly surpasses the typical human sweat rate and validates that the wicking rate of the paperfluidic can directly correspond to the sweat loss volume. Hence, in a typical sweat induction experimental scenario, the fluid front can be converted to understand sweat loss volume, and by incorporating the sweating time, the sweat rate can be readily computable without the requirement of imaging. A proof-of-concept in situ experimentation validated this and yielded a typically expected sweating rate from a less intense exercise session (Supplementary Video SI: 6:35). 

### 3.4. Optical Sweat Biochemistry Analysis 

**Figure 5:**
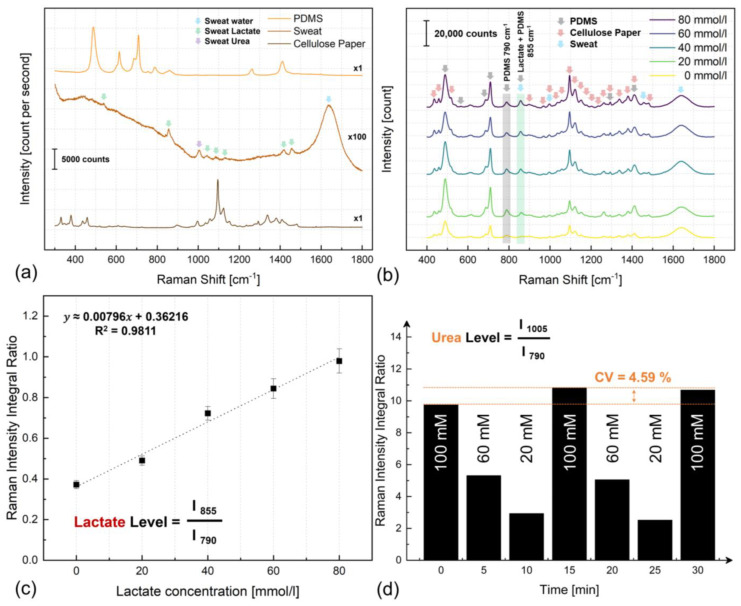
The bioreceptor- and label-free optical Raman scattering biochemical sensing (Raman biosensing) application in sweat analysis utilizing proposed paper-based soft optofluidics for swift sweat collection and transportation. (**a**) Raman scattering spectra from each component of the soft microfluidic platform separately. (**b**) The acquired ex vivo Raman spectra (unprocessed), obtained under dynamic conditions, emerge as a composite of PDMS, sweat, and cellulose contributions. Distinct small-scale Raman bands associated with sweat are discernible. Offsets were added for clarity. (**c**) The calibration curve was derived from the analysis of sweat lactate content. (**d**) Urea biosensing during dynamic in-flow measurements.

In addition to the sweat rate estimation, the paper-based soft microfluidic system assesses the concentration of sweat analytes without using any receptors by label-free Raman biosensing. By directing the Raman laser beam onto the surface of the microfluidic channel (Figure 1c), the resulting Raman signal encompasses prominent Raman bands associated with cover layer material (i.e., PDMS, Supplementary Table S3) alongside the Raman shifts characteristic of cellulose components of the channel layer (Supplementary Table S4). The configuration depicted in Figure 5a shows the Raman bands originating from pristine PDMS, sweat samples obtained in controlled in vitro conditions on a glass microscope slide, and congested Raman shifts attributed to the cellulose composition.

Given that the Raman spectral intensity of liquid sweat is approximately two orders of magnitude weaker than that of cellulose and PDMS Raman responses, their superimposition results in the dominance of Raman bands associated with PDMS or cellulose bands rather than sweat components. Nevertheless, it is still feasible to identify bands related to lactate, which remain unaffected by the substantial Raman activity of the cellulose paper substrate—specifically, the lactate peaks at 855 and 1457 cm^−1^. While the former band coincides with a less intense Raman activity of PDMS at 857 cm^−1^, the latter lactate-associated band remains unobstructed by PDMS interference. In an ideal scenario, if the latter lactate-associated band exhibited an intense presence, it could be harnessed for quantifying lactate levels. However, our prior investigations have demonstrated that the biosensing utilizing the former lactate band exhibits a nearly 100% higher sensitivity than the latter [7]. In addition, during experimentation with paperfluidics, we encountered limitations in utilizing the latter band to accurately assess physiological lactate concentration levels below 40 mmol/L. Hence, the sole avenue for lactate level analysis utilizing the Raman-paper fluidics platform lies in utilizing the 855 cm^−1^ band, which, in this instance, is inevitably subject to interference from one of the small Raman bands of PDMS. 

To mitigate the interference issue, this study introduces a ratiometric approach to remove the impact of PDMS-induced intensity interference on the lactate band. PDMS exhibits high Raman transparency and minimal thickness inhomogeneity due to the blade casting fabrication method, resulting in negligible variations in PDMS thickness-related interference from one point to another. However, considering variations in laser focus is crucial as they impact both PDMS and the bands associated with sweat analytes. Therefore, employing ratiometric analysis is integral to accounting for and neutralizing potential discrepancies stemming from these laser focus variations to sweat analyte concentration. 

Figure 5b presents the Raman spectra acquired ex vivo, employing a porcine skin phantom as an optimal human epidermis mimic. As expected, the resultant Raman spectra are congested, encompassing contributions from all constituents within the paper-based microfluidic system during sweat operation. However, we show that quantification of the lactate level could be achieved by calculating the ratio of the integrated area under the curve (AUC) of the peak at 855 cm^−1^ (combining lactate and PDMS) to any PDMS-specific peak, such as the one at 790 cm^−1^ (solely attributed to PDMS). This ratio offers a quantifiable measure associated with lactate concentration in sweat. Figure 5c demonstrates the calibration curve from this ratiometric analysis, encompassing lactate concentration levels from 0 to 100 mmol/L, which presents linear behavior, evidenced by an R^2^ value of 98.11%. 

Given the nature of this analysis being ratiometric, the sensitivity measurement presented herein differs from the conventional count/mM unit typically employed in Raman biosensing [7,63]. Meanwhile, the laser focal point remains positioned above the surface of the phantom skin, effectively obstructing any substantial penetration of its light into the skin tissue. This outcome proves advantageous, as no Raman activity from the porcine skin is detected. This specific outcome aligns with our intent, as the absence of any epidermal indications signifies the successful blockage of the laser beam from reaching the skin surface.

To further demonstrate the continuous, in-flow, dynamic biosensing capabilities of the paper-based microfluidic system and underscore its potential for analyzing additional sweat analytes, we conducted another ex vivo experiment, this time by adding an outlet reservoir and performed sweat urea biosensing. Figure 5d shows ratiometric urea biosensing during ex vivo continuous in-flow dynamic measurements. A discernible Raman band centered around 1005 cm^−1^ manifests alterations in its intensity, correlated with changes in a concentration ranging from 100 mmol/L to 60 mmol/L and eventually to 20 mmol/L in a time frame of 30 min experiment (Supplementary Figure S4). This dynamic experimentation establishes the viability of sweat urea biosensing using the proposed merger of Raman spectroscopy with paper-based optofluidics and underscores its high repeatability, as demonstrated by a maximum CV of 4.59%.

## 4. Discussion

In this study, we developed a paper-based optofluidics for sweat analysis using Raman scattering, particularly in exercise-induced perspiration. However, the versatility of this platform extends beyond exercise-induced sweat and can be readily adapted for the analysis of sweat induced by thermal, artificial, or stress/anxiety factors. Furthermore, given that the paperfluidic system capitalizes on the fundamental wicking properties of porous cellulose-based materials without requiring active or passive pumping, its applicability can be extended to various biofluid analyses, such as urine or saliva. In such scenarios, it is necessary to modify the packaging to facilitate swab sampling and in vitro diagnostics, akin to the ex vivo lactate level experimentation presented in this investigation. The selection of channel size—be it long or short—depends on the intended application. For our study, monitoring sweat rate during exercise demands a larger channel to effectively accommodate sweat secretion. A shorter channel would lead to saturation, filling the entire channel with sweat and hindering accurate estimation of sweat rate. 

On the other hand, the material choice for the channel of the paperfluidics platform necessitated careful consideration of multiple factors, including material selection for each layer, the need for an encapsulator, and various channel geometrical parameters such as width, length, and inlet diameter. In selecting the channel layer material, we explored alternatives beyond grade 1 filter paper, including grade 4, 113, and chromatographic paper. Interestingly, we discovered that the suitability of the material for sweat rate experiments did not solely determine the choice. The aforementioned paper types proved viable options for this application (Supplementary Materials Note S3: Wicking Rate Simulation and Estimation). 

However, we excluded two options from further consideration due to their larger thickness, which diminishes the precision regarding sweat rate estimation and the difficulty of patterning those substrates by the in-used tool [64]. Consequently, grades 4 and 113 were eliminated from consideration, leading to the choice between grade 1 and chromatographic paper. During the optical biosensing experiments, we observed that chromatographic paper yielded less inter-device repeatability than grade 1 paper, possibly due to its higher degree of uniformity. As a result, the grade 1 paper was selected as the material of choice. Additionally, we found that including a cover layer encapsulator was necessary to prevent liquid evaporation, especially in outdoor sweat collection experiments. We considered three viable cover-layer options: Kapton polyimide (PI), PET, and PDMS. In our experiments, PDMS exhibited lower Raman activity than the others, leading to our decision to proceed with PDMS as the encapsulator [45]. 

Moreover, we matched the inlet diameter and channel width to avoid dead volume creation and accommodation. Also, the inlet and outlet diameters were matched to avoid back pressure. Utilizing channel widths greater than 2 mm would indeed enable the incorporation of more sweat; however, this would be counterbalanced by the accompanying increase in channel width. Consequently, the delay in the arrival of the first sweat sample at the biosensing spot did not significantly differ when weighed against the advantages of employing larger inlet sizes to encompass a broader range of sweat pores and acquire larger sweat volumes.

We have also successfully designed and tested devices with 1 mm channel widths. However, we encountered significant difficulties with these narrower channels during developing and constructing the paper-based optofluidic units. Consequently, we decided to proceed exclusively with 2 mm channel widths for continued development and experimentation. Regarding the channel length, the decision primarily hinges on the specific application requirements where more extended channels facilitate the analysis of the average sweat rate over extended sweating sessions.

Certainly, while sweat loss quantification and sweat rate estimation in this study are not conducted through Raman spectroscopy, there is a potential avenue for “sensing” sweat rate by employing a more complex Raman system instrumentation. This possibility involves using two Raman sensing spots within the paperfluidics. 

By enabling online, real-time data processing, the elapsed time between changes in analyte concentration recorded at spot 1 (an earlier location in the channel) and spot 2 (a farther location in the channel) could potentially indicate sweat rate. However, it is important to note that this approach would necessitate more intricate instrumentation, involving the incorporation of directing the Raman laser to two sensing spots and real-time data processing capabilities. In this study, we opted for sweat loss quantification and the estimation of sweat rate based solely on visual analysis due to its practicality within the scope and objectives of our research.

While the bench-top unit offers the advantage of customizable measurement settings, providing fine spectral resolution and high sensitivity, we intentionally refrained from utilizing these capabilities. Instead, we tailored our experimental Raman parameters to mirror the capabilities of established commercially available portable/hand-held Raman devices. This deliberate approach aimed to replicate the technological characteristics of such devices to ensure alignment in our data collection methodologies. However, as part of the future directions for this study, we plan to develop a customized portable system for further assessment of our recordings and perform real-life on-body experimentation. The challenge in transitioning to such Raman systems will not primarily revolve around recording resolution and acquisition parameters. Instead, the challenge rests in experimental difficulties and intricacies in adjusting the laser focal point and maintaining its consistency during measurements. Past studies have reported ratiometric analysis to suppress the 60% reached variations in intensity caused by laser point variation of ±2 mm [17]. Indeed, as extensively outlined in the limitations section (Supplementary Materials Note S3: Limitations), the fundamental challenge in merging paper-based microfluidic devices with any Raman scattering recording technique centers around the consistent maintenance of the laser focal point. Even smaller movements can induce considerable alterations in spectra and associated intensities. Our investigations have shown that the corrections facilitated by ratiometric analysis alone did not consistently yield repeatable results. Therefore, we made a concerted effort to re-adjust the laser focal point before each measurement.

The efficiency of liquid transportation through porous materials of the presented paperfluidics is shown to boast a fivefold improvement compared to soft epidermal microfluidic systems comprised of plastics or other soft materials. In addition, the capacity of the system to monitor sweat rate by quantifying the wicking rate of cellulose paper through visual inspection obviates the need for imaging or any onboard electronic components. This feature simplifies the acquisition of sweat rate data for skin-interfaced biosensing devices to enable accurate sweat biochemical analysis. Furthermore, the inherent versatility of the paper material facilitates pattern creation with remarkable ease, surpassing the capabilities of other materials and simplifying the fabrication process while maintaining sustainability. Additionally, its natural hydrophilic properties contribute to leak prevention, a concern mainly reported with materials like PDMS. 

The limitations of this study are further discussed in the Supplementary Materials Note S4: Limitations. 

## 5. Conclusions

This study presents a wearable epidermal opto-paperfluidic device for Raman spectroscopy-assisted sweat biochemical sensing (Raman biosensing). The system has a slender profile, measuring 0.36 mm in height and weighing a mere 0.19 g, occupying a compact area of only 3 cm^2^. The wearable component of the reported device pertains to the soft microfluidic section, while the Raman spectroscopy segment responsible for optical biosensing currently lacks the wearable attribute. As a great advantage, the biochemical analysis shown in this study is entirely free of nanoparticles, mediators, membranes, biorecognition or bioreceptor elements (e.g., enzymes, antibodies, aptamers, molecularly imprinted polymers), plasmonic (meta)surfaces, and integrated transducers or electronic components within the soft epidermal microfluidic platform. 

The channel layer of the device is designed in a serpentine-like shape and optimized through mechanical simulations to withstand natural skin deformations. Later, we elucidated the stepwise process of developing this unit through a sustainable process using elementary low-cost tools. Next, we rigorously assess the mechanical robustness of the device through extensive in vitro and in situ experiments involving bending, compression, stretching, and twisting. Subsequently, we empirically study the pivotal aspect of sweat rate data in sweat analysis and its impact on sweat component levels employing Raman spectroscopy and sampling exercise-induced sweat. In addition, we show the feasibility of using fluid front to quantify the wicking rate and correlate it with sweat loss volume. Indeed, the fluid front position (designating fluid travel distance) is linearly correlated with an R^2^ of 99.29% with fluid volume with a sensitivity of 3.48 μL/mm. Furthermore, through ex vivo experiments featuring synthetic sweat containing over 30 analytes and porcine skin phantom, which is a close approximation to human skin, the device enabled bioreceptor-free, simultaneous, and multiplexed monitoring of sweat lactate and urea concentrations within dynamically flowing sweat using highly selective ratiometric quantitative Raman spectroscopy analysis. 

In summary, the fusion of Raman spectroscopy, a potent bioreceptor-less and label-free analytical tool, with the distinctive attributes of low-cost cellulose-based soft microfluidic platforms, “Raman–paperfluidics”, holds great promise to serve as a pivotal facilitator in sustainable point-of-care or point-of-need chemical analysis, especially in resource-limited settings, from low-volume liquid samples, in case of medical specimens where the samples hold substantial rarity and cost, as well as in the context of forensic traces and pharmaceutical compounds. 

## Data Availability

Data are contained within the article or supplementary files.

## Supplementary Materials

The following supporting information [2,7,17,28,34,45,65,66,67,68,69,70,71,72,73,74,75,76,77,78,79,80,81,82,83,84,85,86,87,88,89,90,91,92,93] can be downloaded at: https://www.mdpi.com/article/10.3390/bios14010012/s1.
