# Supplementary material for: Soft Epidermal Paperfluidics for Sweat Analysis by Ratiometric Raman Spectroscopy
Source: Biosensors (Basel). 2023 Dec 25;14(1):12. doi: 10.3390/bios14010012 (PMC10812966; doi:10.3390/bios14010012)
Supplement: Supplementary file 1 [file biosensors-14-00012-s001.zip › biosensors-2753366-supplementary.pdf]

Supplementary Materials for

Soft Epidermal Paperfluidics for Sweat Analysis by Ratiometric Raman Spectroscopy

Ata Golparvar [1]*, Lucie Thenot [1], Assim Boukhayma [2] and Sandro Carrara [1]

[1] Bio/CMOS Interfaces (BCI) Laboratory, École Polytechnique Fédérale de Lausanne (EPFL), 2000 Neuchatel, Switzerland
[2] Senbiosys SA, 2000 Neuchatel, Switzerland
* Correspondence: Corresponding Author (ata.golparvar@epfl.ch)

Contents

Supplementary Videos 2

Supplementary Tables 3

Supplementary Figures 5

Supplementary Notes 8

Note 1: Significance of Sweat Urea and Lactate Analysis 7

Note 2: Materials and Methods 8

A. Chemicals and Reagents 8

B. Optical Biosensing Measurements 8

C. Data Analysis 9

Note 3: Wicking Rate Simulation and Estimation 10

Note 4: Limitations 12

References 13

Supplementary Video SI.

The video showcases the straightforward development process of developing the opto-paperfluidic unit and its deployment on the human arm. The simple construction of the paperfluidic unit is accomplished within less than five minutes, employing elementary tools. The structure involves the incorporation of three primary layers. The first "base layer" is a double-sided adhesive tape that is initially enveloped by two protective cover layers that can be conveniently detached from them during the fabrication process. The intermediary layer is the patterned Whatman filter paper. The third topmost layer comprises a 100 μm thick polydimethylsiloxane (PDMS) sheet, which is initially sandwiched between easily detachable polyethylene terephthalate (PET) layers. An additional layer to stop laser penetration may be integrated if high laser powers are used. To fabricate the paperfluidic unit, first, the previously patterned Whatman filter paper is positioned on a commercially available double-sided irritative-free adhesive tape. Secondly, the designated input and output regions of the soft microfluidic chip are marked: input on the tape and output on the PDMS sheet. Third, the marked spots are pierced by a cost-effective, hand-held instrument purchased from a local hardware store. Subsequently, the protective PET shielding layer enwrapping the adhesive tape is removed, thus allowing for the secure adhesion of the filter paper onto the exposed adhesive substrate. Consecutively, the PET covering enveloping the PDMS sheet is detached, thereby facilitating the positioning of the PDMS layer on top of the already attached patterned paper channel and adhesive layer. This sequence step finalized the assembly process of the paperfluidic unit, rendering it available for immediate operational deployment. Continuing the video, the protective PET layer encapsulating the PDMS sheet is detached, followed by the separation of the white tape holder. Later, the now fully exposed paperfluidic unit is affixed to the forearm of a volunteer participant. The video highlights the vigorous twisting and stretching of the paperfluidic unit while attached to a human arm. Finally, the video concludes with a photograph taken after a successful sweat collection during a brief exercise session.

Supplementary Video SII.

The video features the exposed paperfluidic unit while it was subjected to stretching and bending tests, where the developed unit successfully withstands the experimental conditions. Subsequently, the video proceeds to depict a series of experiments involving stretching and compressing the paperfluidic unit while mounted on a human arm. In the second phase, the paperfluidic unit is colored with a vivid pink colorant and has an additional 110 μm thick aluminum tape laser-blocking layer.

Supplementary Video SIII.

The video presents the paperfluidic wicking process of a 20 μL droplet over ~5 minutes. The fluid front position is a visually measurable indicator of droplet volume intake, which represents sweat loss during sweating sessions and can be related to sweat rate without imaging solely by visual inspection.

Table S1. Peak assignment for the Raman shifts of the aqueous lactate solution. The vivid shifts of lactate, even among the congested Raman bands of the cellulose paper and PDMS sheet, are highlighted. Incorporated with another reference band (such as one of the intense PDMS peaks), these bands can be used in ratiometric lactate level biosensing. This study uses the lactate band at 855 cm^{-1}, since it is more intense [1].

Lactate Raman band [cm^{-1}]	Peak Assignment [2]
535	CO_2^- wagging
780	CO_2^- scissoring
855	C-CO_2^- stretching
925	CH_3 rocking
1045	C-CH_3 stretching
1085	CO stretching
1127	CH_3 rocking and CO stretching
1322	CH scissoring
1365	CH_3 symmetric scissoring,
1418	CO_2^- symmetric stretching
1457	CH_3 asymmetric scissoring

Table S2. Peak assignment for the Raman shifts of the aqueous urea solution. The vivid shift of urea, even among the congested Raman bands of the cellulose paper and PDMS sheet, is highlighted. Incorporated with another reference band (such as one of the intense PDMS peaks), the band at 1005 cm^{-1} can be used in ratiometric urea level biosensing.

Urea Raman band [cm^{-1}]	Peak Assignment [3]
1005	N-C-N stretching
1160	NH_2 rocking

Table S3. Peak assignment for the primary Raman shifts of the developed PDMS sheet. The highlighted band is the in-used reference Raman shift employed in the presented ratiometric analysis to quantify sweat urea and lactate levels in this paperfluidics.

PDMS Raman band [cm^{-1}]	Peak Assignment [4]
490	Si-O-Si stretch
614	NA
708	Si-CH_3 symmetric rocking
753	Si-C symmetric stretching
790	CH_3 asymmetric rocking and Si-C asymmetric stretching
857	CH_3 symmetric rocking
1263	CH_3 symmetric bending
1411	CH_3 asymmetric bending

NA: Not available/unknown.

Table S4. Peak assignment for the primary Raman shifts of the cellulose-based Whatman filter paper.

Paper Raman band [cm^{-1}]	Peak Assignment [5]
330	Some heavy atom bending
379	Some heavy atom stretching
437	Some heavy atom stretching
458	Some heavy atom stretching
894	HCC and HCO bending
996	stretching
1034	stretching
1056	stretching
1096	stretching
1124	stretching
1152	Some heavy atom stretching
1292	HCC and HCO bending
1337	HCC, HCO, and HOC bending
1379	HCC, HCO, and HOC bending
1408	HCC, HCO, and HOC bending
1480	HCH and HOC bending

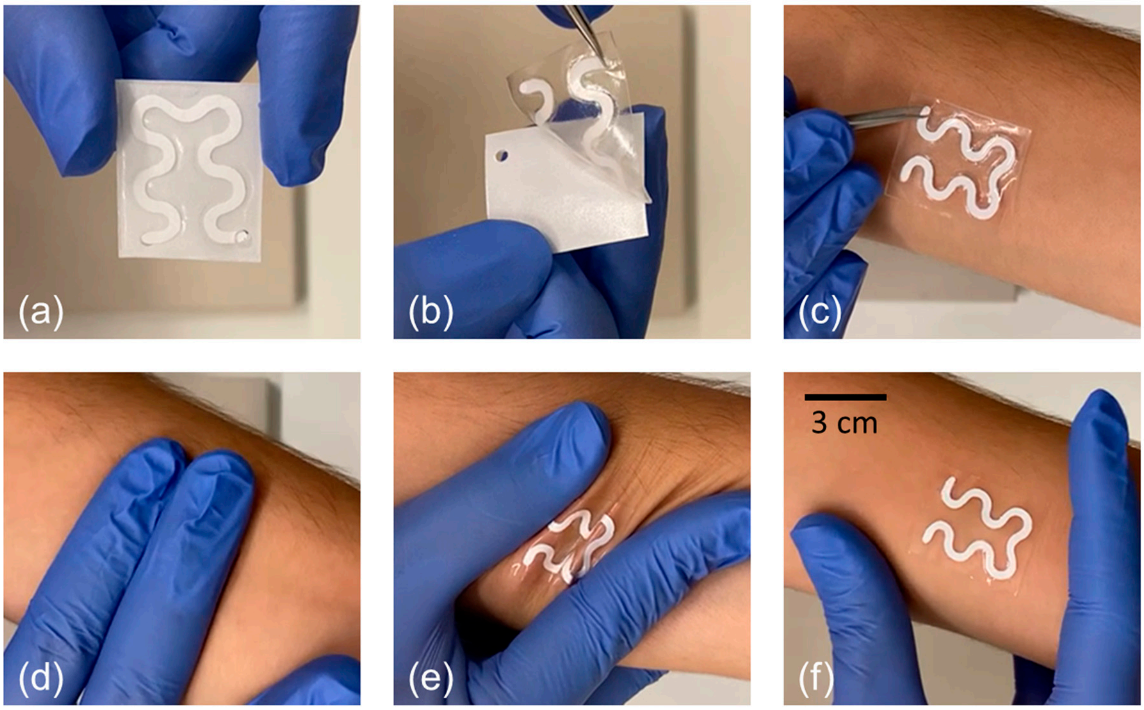

Figure S1. Assessment of skin conformability and mechanical integrity in the developed paperfluidic unit. (a) The fabricated paperfluidic unit comprises a serpentine paper-based channel enveloped between a medical-grade irritation-free adhesive and PDMS layers. In addition, a white protective layer beneath the adhesive safeguards the adhesive-tape interface prior to its affixation onto the skin, and a PET layer situated above the PDMS layer mitigates dust accumulation on the PDMS surface. The application process involves the sequential removal of the (b) white adhesive protective layer and the PET cover of the PDMS layer, followed by (c) the placement of the soft microfluidic unit at the desired body site. (d) The subsequent application of gentle pressure facilitates the secure and conformal attachment of the unit onto the epidermis. (e) An evaluative mechanical deformation test, confirming the paperfluidic resilience and structural integrity as evidenced by (f) its successful endurance in the demonstration tests.

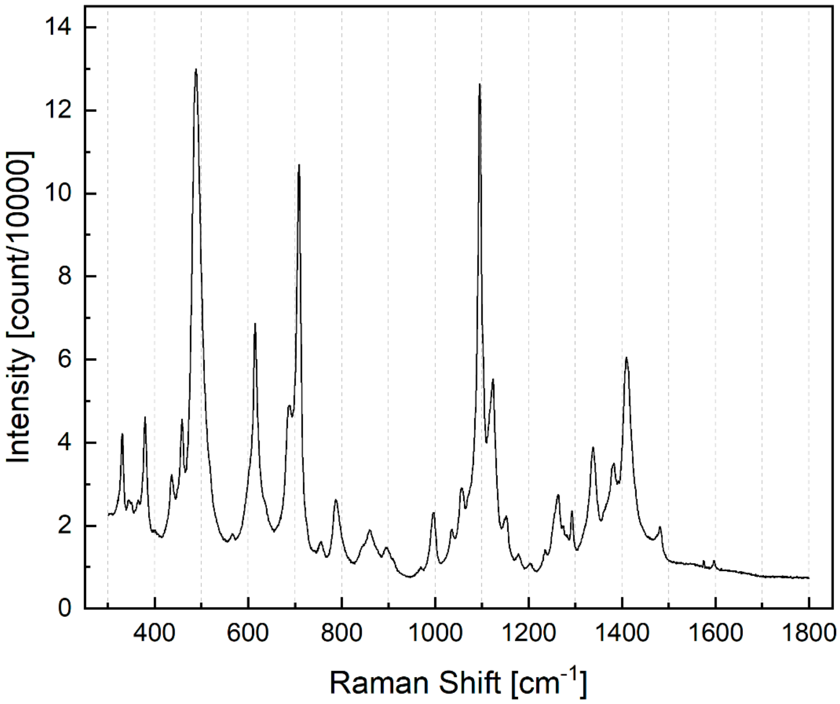

Figure S2. The Raman spectra obtained from the paperfluidic channel without sweat display a congested spectral profile, showcasing a superposition of Raman bands associated with both the PDMS cover and the cellulose channel layers.

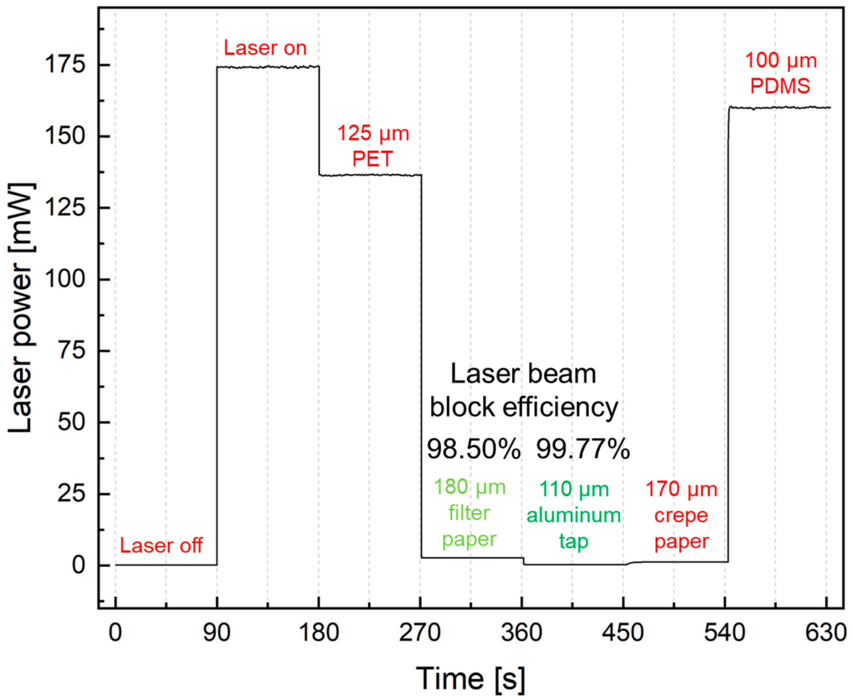

Figure S3. Characterization of the different materials such as PET, Whatman grade 1 filter paper, aluminum tape, black crepe paper, and PDMS as possible laser blockers to completely isolate the skin from laser radiation. In the first 90 s, the laser was turned off. In the next 90 s, the 532 nm laser was turned on without any blocker, and then different laser blocker layers were inserted between the laser and light detector. Aluminum tape blocks almost 100% of the laser power, while the filter paper effectively blocks 98.5% of the incoming laser beam. A portable Starlite power meter (Starlite, Ophir Optronics, Israel) set to the 532 nm beam input was employed for the laser blocking test, with data recorded without averaging.

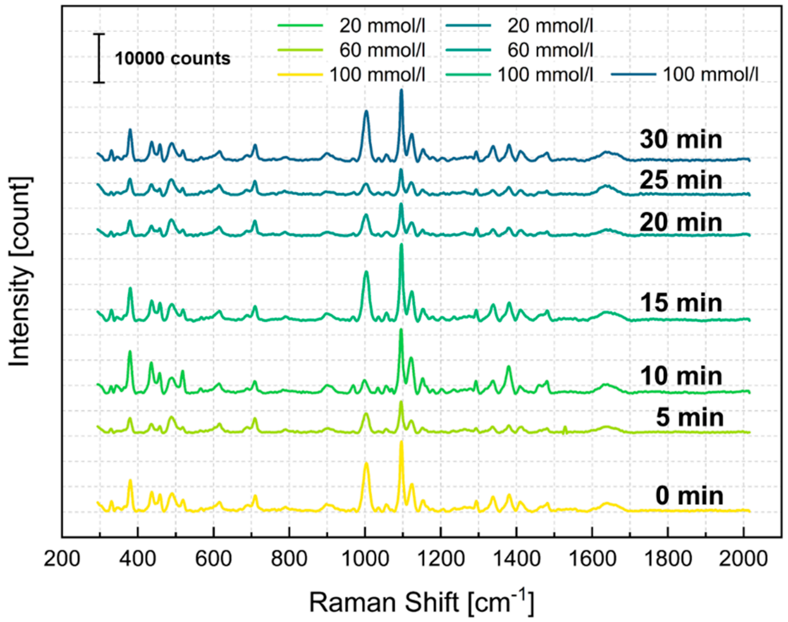

Figure S4. Processed Raman spectra, subject to baseline removal and smoothing, were acquired during the dynamic measurement employing the paper-based optofluidic unit. In this experiment, an auxiliary tissue reservoir at the paperfluidic outlet effectively facilitates sample collection within the channel while sustaining uninterrupted flow, thus averting saturation of the channel layer. The synthetic sweat solution utilized during experimentation spiked with different urea concentrations. Subsequently, the recorded spectra variations associated with the characteristic urea Raman shift around 1005 cm^{-1} were observed.

Note S1: Significance of Sweat Urea and Lactate Analysis

Eccrine sweat urea plays a role as a natural skin moisturizer and functions in excreted metabolic waste, indicating kidney performance (i.e., renal function) [6]. Healthy kidneys remove urea from the body through the glomerular filtration process, but if kidneys fail, the efficiency of this process degrades, and urea concentrations elevate (i.e., uremia). Since variations in plasma urea levels lead to changes in sweat urea due to the diffusive equilibration processes, evidence from multiple groups confirms the correlation between sweat urea and blood urea levels [7–9]. Therefore, urea is the key biomarker for evaluating glomerular operation and is symptomatic of chronic kidney diseases [10]. Moreover, patients with end-stage renal diseases undergo dialysis and require appropriate timing and duration, which can be achieved by continuous urea monitoring [10]. Furthermore, low urea levels indicate complications such as decreased metabolic activity, malnutrition due to a low-protein diet, and liver diseases [11].

Similarly, eccrine sweat lactate plays a role as a natural skin moisturizer [12]. In addition, lactate is a gluconeogenic substrate and functions as a cell signaling molecule [13]. More importantly, sweat lactate level is connected to exercise intensity and, therefore, could be used as a proxy to tedious blood lactate measurements during field and lab training sessions to trace effort and determine the lactate threshold of elite athletes [14]. Although tangible controversy exists on whether sweat lactate correlates to blood lactate, recent studies provide hard evidence of such correlation [15,16]. In particular, the change in lactate production from muscle cells has been demonstrated to induce a simultaneous change in the blood through autonomic nervous balance, hormones, acid−base equilibrium, and metabolic dynamics [17].

Note S2: Materials and Methods

A. Chemicals and Reagents

Analytical-grade powders of l-lactate ($C_3H_5NaO_3$, 98%) and urea (CH_4N_2O, 98%) were purchased from Sigma-Aldrich (MilliporeSigma, USA) and employed as received without further purification. Artificial sweat was obtained from Biochemazone (BZ 119, Chemazone, Canada). This synthetic sweat closely emulates human eccrine sweat with an adjusted pH of 6.3 and comprising a comprehensive composition, including 19 amino acids (namely glycine, l-alanine, l-arginine, l-asparagine, l-aspartic acid, l-citrulline, l-glutamic acid, l-histidine, l-isoleucine, l-leucine, l-lysine as hydrochloride, l-methionine, l-ornithine as hydrochloride, l-phenylalanine, l-serine, l-threonine, l-tyrosine, l-valine, and taurine), 7 minerals (namely calcium, chloride, iron, magnesium, potassium, sodium, sulfate, and zinc), and 5 key metabolites (namely ammonia, glucose, lactate, urea, and uric acid). Aqueous solutions of lactate and urea were formulated to augment the original synthetic perspiration, aiming to emulate a broad range of sweat metabolite concentrations encompassing both normative (physiological) and perturbed (potentially pathological) scenarios and investigate the sensitivity of the measurements in such states. During the solution preparation process, the powders were weighed using a high-precision scale, dissolved in ultrapure water, subjected to agitation on an orbital shaker, and left refrigerated overnight to attain equilibrium. The solutions were generated employing a type 1 ultrapure water unit (Siemens, Germany), exhibiting a resistance of >18.2 MΩ.cm.

Indoor-exercise-induced sweat was gathered from a healthy volunteer (a 28-year-old male with a BMI of 21.7) subsequent to a 20-minute session of treadmill running set at three distinct speeds (high, moderate, and slow), all conducted within a climate-controlled environment maintained at 298 ± 0.5 K temperature and 42.0 ± 1% relative humidity. The choice of three speeds aimed to induce varying temporal local sweat rate production by modifying exercise intensity [18]. On average, perspiration commenced approximately 5 minutes into the activity, and the process was replicated 3 times on different days, with 1-hour intervals between each collection. For in vitro sweat analysis, sweat was collected with a small cotton-based absorber pad (measuring 3 cm × 3 cm) placed directly on the anterior lower arms for a brief duration of under one minute immediately following the exercise sessions. The selection of the lower arms for sweat collection was guided by their favorable characteristics, including an adequate sweat rate of up to 226 $g.m^{-2}.h^{-1}$ [19], as well as their accessibility without necessitating external assistance. After each collection, the saturated pad was inserted into a 10 mL syringe, and pressure applied to the plunger facilitated the extraction of the absorbed sweat, which was subsequently transferred into a separate container, which, on average, resulted in volumes of approximately 0.3 mL.

A. Optical Biosensing Measurements

Raman spectroscopy was carried out within the spectral range of 350 to 2000 cm^{-1} employing a commercially available backscattered confocal micro-Raman microscopy (LabRAM HR, Horiba, Japan). The spectrometer was configured to a groove density of 600 g/mm. The excitation source was a continuous wave 532 nm single-frequency green laser (Cobolt 05, Hubner Photonics, Germany). The slit width and pinhole diameter were 100 μm and 200 μm, respectively. The objective with 0.55 numerical apertures (NA) was used. The laser power and integration time were 400 mW and 120 s if not otherwise indicated. Throughout the experiments, no photodamage was observed.

Before each measurement session, the spectrometer was calibrated using the reference Raman shift of silicon at 520 cm^{-1}. All the measurements were acquired in a room with 297 K temperature and average daylight. In the context of in vitro measurements, a 100 μl droplet of each human sweat sample was positioned within concave glass microscope slides, including well depths of approximately 800 μm (Electron Microscopy Sciences PA, USA) and laser beam tightly focused to 200 μm below the droplet surface [20]. To evaluate measurement precision, three consecutive spectra were collected utilizing each time a new droplet. For ex vivo measurements, the fabricated flexible paperfluidic unit was affixed to a porcine phantom (purchased from a local butcher).

The porcine phantom is an excellent animal model to emulate the stratum corneum level of human skin [21]. In each iteration, we varied the concentration of one of the two metabolites (urea or lactate) while keeping the concentration of the other analytes constant. We conducted two experiments to explore potential application scenarios with the paper-wicking-based sample loading technique and Raman-scattering-based biosensing. In the initial experiment, we operated the paperfluidic unit without integrating an outlet reserve and performed lactate biosensing. In each iteration, we cast 10 μL of sweat solutions with varying spiked lactate concentrations and repeated the procedure three times for different concentrations. However, since the outlet reserves were absent, the unit was saturated after a while. Therefore, for each solution, a new paperfluidic unit was employed; thus, for analyzing 5 different sweat lactate concentrations, 5 paperfluidic units were used. Inspired by previous studies [22,23], the second experiment introduced a cotton tissue outlet reserve into the setup and carried out urea biosensing in fully dynamic in-flow experimentation. This modification avoided the saturation of the paper channel and enabled continuous measurements of larger volumes. Specifically, in each iteration with sweat samples spiked with different urea levels, we cast 15 μL for each solution.

A. Data Analysis

Data processing was executed offline using Origin software (OriginLab Corporation, MA, USA). An asymmetric least-square fit was applied for baseline subtraction on each spectrum to mitigate the impact of autofluorescence. Parameters for this process included an asymmetric factor and a threshold of 0.001, a smoothing factor of 3, and a total of 10 iterations [24]. Next, we applied a Savitzky–Golay filter with a polynomial order of 3 and a window length of 13 to achieve spectrum smoothing [25]. The absolute area under the 20 cm^{-1} Raman shift bands of interest was integrated for ratiometric data analysis and concentration level prediction. A linear regression fit model was employed to establish the calibration curves and determine measurement sensitivity. The coefficient of variation (CV) was calculated to quantify the variability within the data set. This was achieved by computing the ratio of the standard deviation (σ or STD) to the mean of the n number of samples, presented as a percentage.

Note S3: Wicking Rate Simulation and Estimation

Liquid flow in porous media is governed by two crucial properties: wettability and wicking. In paper-based microfluidics, high wettability at the inlet facilitates liquid uptake when it comes into contact with secreted sweat. This contact causes the porous surface to become wet, thereby enabling the liquid to wick through the porous media. The driving force behind this wicking phenomenon is the negative capillary pressure, which induces the spontaneous flow of liquid within the porous material. The Lucas–Washburn equation, the initial modeling of wicking phenomena, conceptualizes randomly arranged fibers paper as capillary tubes. Indeed, it describes capillary flow in a one-dimensional model in cylindrical tubes. Furthermore, Darcy contributed to understanding wicking in porous media by formulating mathematical models that account for both single-phase and two-phase flows.

To understand and enhance the precision of sweat rate estimation through paper wicking, we conducted FEM simulations with COMSOL Multiphysics. These simulations were instrumental in validating the correspondence between observed wicking absorption and the theoretical predictions outlined by the Lucas–Washburn equation, which effectively estimates the quantity of water absorbed within a medium:

$$L = \sqrt{\frac{\gamma\ r \cos\theta\ t}{2\eta}}$$

L is the traveled distance by water in the paper, γ is the fluid surface tension (here 0.0723[N/m]), *r* is the radius of the pores of the paper (here, we used capillary radius as half of the pore diameter given by the manufacturer, 5.5 µm). θ is the angle of contact (0 here) between the liquid and the solid, *t* is the time, and η is the dynamic viscosity of the fluid (0.001 Pa.s here). The simulation procedure involved modeling a straight paper channel with properties akin to Whatman grade 1 filter paper, possessing dimensions of 120 mm in length and 5 mm in width.

Employing Darcy's law, which elucidates flow within porous mediums, we simulated the fluid dynamics:

$$L = -\frac{k}{\mu}\nabla p$$

L is the traveled distance, k is the permeability of the medium (with porosity of 0.55 $k = 2.0797 \times 10^{-12}\ m^2$), μ is the dynamic viscosity ($\mu_{air} = 1.76 \times 10^{-5}$ and $\mu_{water} = 0.001$ Pa.s), and ∇p is the pressure drop for the given distance. At the base of the paper strip tip, we simulated a continuous influx of water. The outcomes of our simulations (Note 1 Figure) revealed a remarkable alignment with the theoretically predicted Lucas–Washburn formula. Discrepancies observed towards the end of the curves may arise from minor alterations in any of the parameters, given that the Lucas–Washburn formula involves simple multiplicative/divisive relationships between them.

To validate our simulations, a series of experiments were executed. The objectives of these experiments were to ascertain the porosity levels and wicking rates across various cellulose-based papers, specifically Whatman grade 1, 4, and 113 qualitative filter papers, in addition to grade 3MM Chr cellulose chromatography paper. The experimental protocol unfolded as follows: paper channels of 1, 2, 3, 4, and 5 mm in width and 100 mm in length were precisely cut using a lever-action cutter. Length measurements were recorded employing a Vernier Caliper. Subsequently, the paper strips were horizontally arranged within a clean plastic container. Varying quantities of water, ranging from 5 µl to 30 µl, were accurately dispensed using precision pipettes onto the plastic container. The tip of the paper channels was gently maneuvered towards the water droplet. The entire experiment was documented via video recording utilizing an iPhone X camera to extract fluid front position later. Experimental determination of the wicking rates for each type of paper was facilitated through analysis of the video recordings. The findings demonstrated that these papers exhibited wicking rates considerably surpassing the sweat rate observed

on the forearm of a young male human, which typically ranges around 181 g/m²/h [26]. In contrast, the paper wicking rates fell within the 1256 to 3845 g/m²/h range. During these experiments, the paper strips were not enclosed within a PDMS (polydimethylsiloxane) cover, which potentially has a cover layer, leading to an even higher wicking rate due to the removal of evaporation influence.

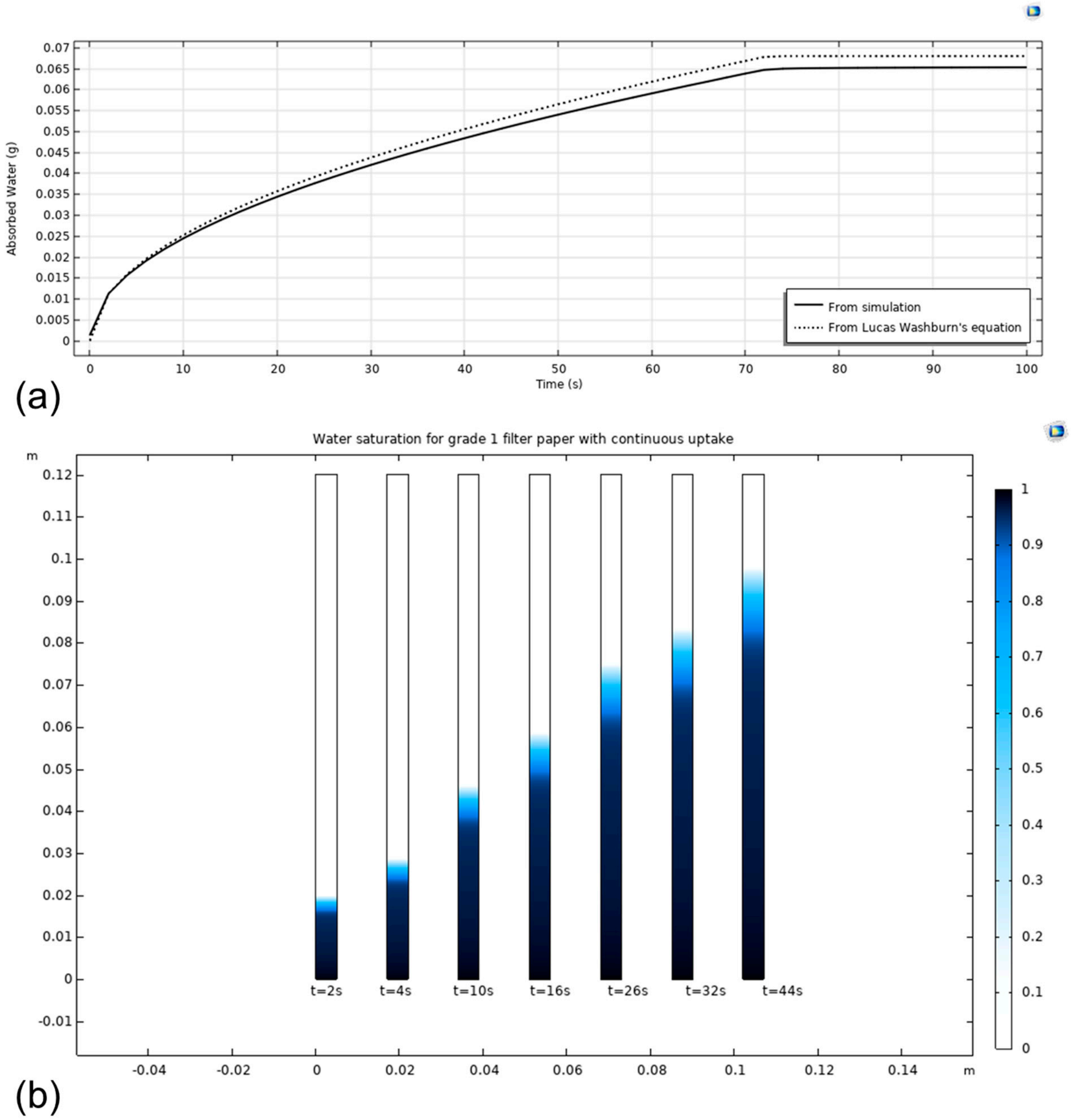

Note 1 Figure. (a) Theoretical liquid travel distance prediction by Lucas–Washburn equation compared to the simulated water uptake governed by Darcy's law. (b) Wicking simulation with grade 1 filter paper showing water saturation levels in different durations.

Note S4: Limitations

As a great advantage, the optical biosensing utilized here exhibits a higher degree of environmental sustainability when compared to alternative biosensing methodologies reliant on enzymes or antibodies. Additionally, the fabrication process outlined here, other than being facile and cost-effective, is fully sustainable. Nonetheless, this paper-based microfluidic unit does not meet complete biodegradability standards. This partial non-biodegradability stems from including base adhesive and the cover PDMS layers, while the cellulose-based material employed for the channel layer imparts an ecologically friendly characteristic. However, adopting commercially available biodegradable double-sided, irritation-free adhesives remains feasible, contingent only on resource allocation. Nonetheless, the cover PDMS layer material is non-biodegradable by nature, but it can undergo absorption in wastewater treatment facilities, with its degradation catalyzed by various clay-based materials, thereby mitigating its long-term environmental impact [27]. Continuing this work, we are identifying a suitable replacement for PDMS, aiming to develop a fully biodegradable, home-compostable, soft microfluidic platform.

A notable concern in our study revolves around potential challenges in measuring sweating time at the onset of perspiration. Our methodology relies on relating the extent of liquid wicking on the paper to sweat lost volume. This approach necessitates knowledge of the sweating time to derive the sweat rate, which signifies the amount of sweat lost over the sweating duration. However, determining sweating time remains a hurdle within our current procedures. In our in situ experiments, we inferred the initiation of sweating by observing liquid wicking at the paperfluidic inlet, thereby assigning sweating time. Looking ahead, the complete miniaturization and integration of our system into a wearable unit may offer a potential solution: if the sensing spot closely aligns with the paperfluidic inlet in this future setup, Raman data could potentially signal the arrival of the sweat sample at that location, effectively indicating the onset of sweating.

In addition, the optical biosensing measurements in this study employed a bench-top micro-Raman spectroscopy instrument, departing from the more commonly favored hand-held or portable instruments typically used in similar investigations [28–30].

On the other hand, the soft optofluidic unit developed in this study is ideally tailored for single-use applications. This primarily arises from the non-reusability of the commercial adhesive interface upon contact with the skin. However, the paper-based channel layer can be efficiently cleaned and reinstated for use through a straightforward washing process. This procedure entails introducing a common cotton tissue into the outlet, rinsing the input with water, and allowing it to dry for subsequent applications. In the future of this work, we will explore alternative adhesive solutions, aiming to enable multiple uses. One promising avenue involves incorporating materials such as silk sheets fabricated from Bombyx mori cocoons, which provide adhesive-less, conformal skin attachment, as demonstrated in our previous work [31]. Nevertheless, as part of our resource conservation efforts, we conducted successful experiments involving the multiple usages of this paperfluidics (a maximum of three times) by gently detaching and reattaching the device.

Furthermore, here, we have shown the first application of integration of paper-based optofluidics with spontaneous Raman spectroscopy without using plasmonic surfaces. This is primarily due to the higher concentration of the target analytes, namely sweat urea, and lactate, compared to other micromolar-ranged sweat analytes, such as uric acid, which can only be detected by enhanced Raman spectroscopy methods [23]. However, apart from the inherent complexities associated with the fabrication of plasmonic surfaces designed for localized near-filed enhancement, it has been widely acknowledged that such systems exhibit limited measurement repeatability [32]. This repeatability issue is further exacerbated in the case of paper-based substrates, primarily due to substrate uniformity [33]. Henceforth, in the present study, we demonstrate that for analyzing sweat components such as urea and lactate, the reliance on plasmonic field enhancement is dispensable, and spontaneous (non-enhanced) Raman spectroscopy yields accurate, repeatable results.

Finally, when comparing our paper-based optofluidic system to other soft microfluidic platforms, such as those composed of PET or PDMS, we find that it imposes more stringent requirements on laser focusing. Indeed, in our prior study utilizing these materials [1,34], we recorded a substantial margin of error in laser focal positioning, allowing deviations of up to ± 100 μm from the midpoint of the channel. However, in our present work, the paramount concern is maintaining laser focus at a stable position. For example, paper swelling occurs during sweat wicking due to the expansion of the interfiber and interfiber pores of cellulose, which inevitably leads to alterations in the laser focal spot. Nonetheless, the laser focus requirements in this work are still comparatively less demanding when contrasted with plasmonic paper-based Raman microfluidics [23,35,36]. In the latter, the need for meticulous control over the laser focus point, ensuring precise alignment within designated plasmonic "hot spots," inherently leads to much more metrological challenges.

References

[1] A. Golparvar, J. Kim, A. Boukhayma, D. Briand, S. Carrara, "Highly accurate multimodal monitoring of lactate and urea in sweat by soft epidermal optofluidics with single-band Raman scattering," *Sensors and Actuators B: Chemical*, vol. 387, p. 133814, 2023. https://doi.org/10.1016/j.snb.2023.133814

[2] G. Cassanas, M. Morssli, E. Fabregue, L. Bardet, "Vibrational spectra of lactic acid and lactates," *Journal of Raman spectroscopy*, vol. 22, pp. 409-413, 1991. https://doi.org/10.1002/jrs.1250220709

[3] R. Keuleers, H. Desseyn, B. Rousseau, C. Van Alsenoy, "Vibrational analysis of urea," *The Journal of Physical Chemistry A*, vol. 103, pp. 4621-4630, 1999. https://doi.org/10.1021/jp984180z

[4] D. Cai, A. Neyer, R. Kuckuk, H. M. Heise, "Raman, mid-infrared, near-infrared and ultraviolet–visible spectroscopy of PDMS silicone rubber for characterization of polymer optical waveguide materials," *Journal of Molecular Structure*, vol. 976, pp. 274-281, 2010. https://doi.org/10.1016/j.molstruc.2010.03.054

[5] J. H. Wiley, R. H. Atalla, "Band assignments in the Raman spectra of celluloses," *Carbohydrate Research*, vol. 160, pp. 113-129, 1987. https://doi.org/10.1016/0008-6215(87)80306-3

[6] D. Choi, M. Gonzales, G. B. Kitchen, D. Phan, P. C. Searson, "A capacitive sweat rate sensor for continuous and real-time monitoring of sweat loss," *ACS Sensors*, vol. 5, pp. 3821-3826, 2020. https://doi.org/10.1021/acssensors.0c01219

[7] R. Vanholder, T. Gryp, and G. Glorieux, Urea and chronic kidney disease: the comeback of the century?(in uraemia research)." *Nephrology Dialysis Transplantation* 33.1 (2018), 4-12. https://doi.org/10.1093/ndt/gfx039

[8] R. W. Keller, J. L. Bailey, Y. Wang et al., "Urea transporters and sweat response to uremia." *Physiological reports* 4.11 (2016), e12825. https://doi.org/10.14814/phy2.12825

[9] N. Hanafusa, B. T. Lodebo, A. Shah et al., Hanafusa, Norio, et al. "Is there a role for diaphoresis therapy for advanced chronic kidney disease patients?." *Journal of Renal Nutrition* 27.5 (2017), 295-302. https://doi.org/10.1053/j.jrn.2017.04.008

[10] I. Alvear-Ordenes, D. García-López, J. De Paz et al., "Sweat lactate, ammonia, and urea in rugby players." *International journal of sports medicine* 26.08 (2005), 632-637. https://doi.org/10.1055/s-2004-830380

[11] A. Bonini, F. M. Vivaldi, E. Herrera et al., "A graphenic biosensor for real-time monitoring of urea during dialysis." *IEEE Sensors Journal* 20.9 (2020), 4571-4578. https://doi.org/10.1109/JSEN.2020.2966456

[12] A. Bigot, M. C. Tchan, B. Thoreau et al., "Liver involvement in urea cycle disorders: a review of the literature." *Journal of inherited metabolic disease* 40 (2017), 757-769. https://doi.org/10.1055/s-2004-830380

[13] A. Watabe, T. Sugawara, K. Kikuchi et al., "Sweat constitutes several natural moisturizing factors, lactate, urea, sodium, and potassium." *Journal of dermatological science* 72.2 (2013), 177-182. https://doi.org/10.1016/j.jdermsci.2013.06.005

[14] G. A. Brooks, "The science and translation of lactate shuttle theory." *Cell metabolism* 27.4 (2018), 757-785. https://doi.org/10.1016/j.cmet.2018.03.008

[15] Van Hoovels, Kevin, et al. "Can wearable sweat lactate sensors contribute to sports physiology?." ACS sensors 6.10 (2021), 3496-3508. https://doi.org/10.1021/acssensors.1c01403

[16] W. Gao, S. Emaminejad, HYY Nyein et al., "Fully integrated wearable sensor arrays for multiplexed in situ perspiration analysis." *Nature* 529.7587 (2016), 509-514. https://doi.org/10.1038/nature16521

[17] H. Guan, T. Zhong, H. He et al., "A self-powered wearable sweat-evaporation-biosensing analyzer for building sports big data." *Nano Energy* 59 (2019): 754-761. https://doi.org/10.1016/j.nanoen.2019.03.026

[18] L. Klous, C. De Ruiter, S. Scherrer et al., "The (in) dependency of blood and sweat sodium, chloride, potassium, ammonia, lactate and glucose concentrations during submaximal exercise." E*uropean journal of applied physiology* 121 (2021), 803-816. https://doi.org/10.1007/s00421-020-04562-8

[19] C. J. Smith and G. Havenith, "Body mapping of sweating patterns in male athletes in mild exercise-induced hyperthermia," *European Journal of Applied Physiology*, vol. 111, pp. 1391-1404, 2011. https://doi.org/10.1007/s00421-010-1744-8

[20] A. Golparvar, A. Boukhayma, C. Enz, S. Carrara, "Optimized Detection of Hypoglycemic Glucose Ranges in Human Serum by Raman Spectroscopy with 532 nm Laser Excitation," *Photoptics*, pp. 158-165, 2022. https://doi.org/10.5220/0010981300003121

[21] S. Tfaili, C. Gobinet, G. Josse, J. Angiboust, M. Manfait, O. Piot, "Confocal Raman microspectroscopy for skin characterization: a comparative study between human skin and pig skin," *Analyst*, vol. 137, no. 16, pp. 3673-3682, 2012. https://doi.org/10.1039/C2AN16292J

[22] S. Tonello, T. Fapanni, S. Bonaldo, G. Giorgi, C. Narduzzi, A. Paccagnella, M. Serpellon, "Amperometric measurements by a novel aerosol jet printed flexible sensor for wearable applications," *IEEE Transactions on Instrumentation and Measurement*, vol. 72, 2022. https://doi.org/10.1109/TIM.2022.3225014

[23] U. Mogera, H. Guo, M. Namkoong, M. S. Rahman, T. Nguyen, and L. Tian, "Wearable plasmonic paper–based microfluidics for continuous sweat analysis," vol. 8, 2022. https://doi.org/10.1126/sciadv.abn1736

[24] A. Golparvar, A. Boukhayma, T. Loayza, A. Caizzone, C. Enz, S. Carrara, "Very selective detection of low physiopathological glucose levels by spontaneous Raman spectroscopy with univariate data analysis," *BioNanoScience*, vol. 11, 871-877, 2021. https://doi.org/10.1007/s12668-021-00867-w

[25] N. A. Coull, A. M. West, S. G. Hodder, P. Wheeler, and G. Havenith, "Body mapping of regional sweat distribution in young and older males," *European Journal of Applied Physiology*, vol. 121, pp. 109-125, 2021. https://doi.org/10.1007/s00421-020-04503-5

[26] L. Ceseracciu, J. A. Heredia-Guerrero, S. Dante, A. Athanassiou, I. Bayer, "Robust and biodegradable elastomers based on corn starch and polydimethylsiloxane (PDMS)," *ACS Applied Materials & Interfaces*, vol. 7, pp. 3742-3753, 2015. https://doi.org/10.1021/am508515z

[27] J. Xiao, J. Wang, Y. Luo, T. Xu, and X. Zhang, "Wearable Plasmonic Sweat Biosensor for Acetaminophen Drug Monitoring," *ACS Sensors*, vol. 8, pp. 1766-1773, 2023. https://doi.org/10.1021/acssensors.3c00063

[28] X. He, C. Fan, Y. Luo, T. Xu, X. Zhang, "Flexible microfluidic nanoplasmonic sensors for refreshable and portable recognition of sweat biochemical fingerprint," *npj Flexible Electronics*, vol. 6, 2022. https://doi.org/10.1038/s41528-022-00192-6

[29] H. S. Kim, H. Kim, J. Lee, T. Lee, J. Yun, G. Lee, Y. Hong, "Hand-held Raman spectrometer-based dual detection of creatinine and cortisol in human sweat using silver nanoflakes," *Analytical Chemistry*, vol. 93, pp. 14996-15004, 2021. https://doi.org/10.1021/acs.analchem.1c02496

[30] S. Mirbakht, A. Golparvar, M. Umar, and M. K. Yapici, "Flexible silk-based graphene bioelectronics for wearable multimodal physiological monitoring," *IEEE 36th International Conference on Micro Electro Mechanical Systems (MEMS)*, 2023. https://doi.org/10.1109/MEMS49605.2023.10052459

[31] M. L. Tseng, Y. Jahani, A. Leitis, H. Altug, "Dielectric metasurfaces enabling advanced optical biosensors," *ACS Photonics*, vol. 8, pp. 47-60, 2020. https://doi.org/10.1021/acsphotonics.0c01030

[32] V. Eskandari, H. Sahbafar, L. Zeinalizad, R. Marashipour, and A. Hadi, "A review of paper-based substrates as surface-enhanced Raman spectroscopy (SERS) biosensors and microfluidic paper-based SERS platforms," *Journal of Computational Applied Mechanics*, vol. 53, pp. 142-156, 2022. https://doi.org/10.22059/JCAMECH.2022.322373.705

[33] A. Golparvar, A. Boukhayma, S. Carrara, "Flexible Microfluidics for Raman Measurements on Skin," *IEEE International Symposium on Medical Measurements and Applications (MeMeA)*, 2023. https://doi.org/10.22059/10.1109/MeMeA57477.2023.10171904

[34] S. Lin, X. Lin, S. Han, Y. Liu, W. Hasi, L. Wang, "Flexible fabrication of a paper-fluidic SERS sensor coated with a monolayer of core–shell nanospheres for reliable quantitative SERS measurements," *Analytica Chimica Acta*, vol. 1108, pp. 167-176, 2020. https://doi.org/10.1016/j.aca.2020.02.034

[35] E. P. Hoppmann, W. Y. Wei, and I. M. White, "Inkjet-printed fluidic paper devices for chemical and biological analytics using surface-enhanced Raman spectroscopy," *IEEE Journal of Selected Topics in Quantum Electronics*, vol. 20, pp. 195-204, 2013. https://doi.org/10.1109/JSTQE.2013.2286076
